# Supplementary material for: Sex, Age, and COVID-19 Vaccine Characteristics Associated with Adverse Events After Vaccination and Severity: A Retrospective Analysis
Source: Infect Dis Rep. 2025 Sep 3;17(5):108. doi: 10.3390/idr17050108 (PMC12452524; doi:10.3390/idr17050108)
Supplement: Supplementary file 1 [file idr-17-00108-s001.zip › idr-3761052-supplementary.pdf]

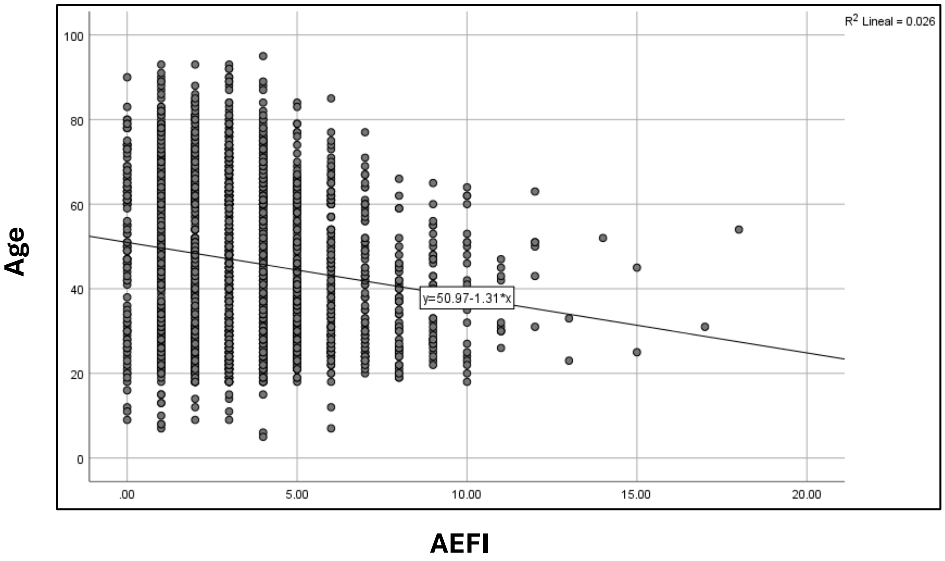

Figure S1. Distribution of vaccine type, age, and adverse events following immunization (AEFI) characteristics among vaccinated individuals

Table S1. AEFI systemic type. State of Nuevo Leon AEFI Notification System, December 2020-June 2022 (n = 2,213).

AEFI systemic type	n	%
Headache	1221	55.20
Myalgia	757	34.20
Fatigue	673	30.40
Fever ≥ 38°C	629	28.40
Bone or join pain	569	25.70
Dizziness	465	21.00
Nausea	462	20.90
Chills	351	15.90
Lethargy	301	13.60
Vomiting	214	9.70
Diarrhea	190	8.60
Odynophagia	138	6.20
Coughing	108	4.90
Abdominal pain	106	4.80
Rhinorrhea	106	4.80
Exanthema	103	4.70
Tachycardia	101	4.60
Pruritus (generalized)	90	4.10
Dyspnea	71	3.20
Edema (generalized)	58	2.60

Irritability	56	2.50
Syncope	35	1.60
Poor appetite	31	1.40
Seizures (with or without fever)	15	0.70
Bronchospasm	14	0.60
Movement limitation	11	0.50
Bleeding	9	0.40
Guillain-Barre syndrome	9	0.40
Paralysis	8	0.40
Thrombocytopenic purpura	6	0.30
Red/itchy eye	4	0.20
Syndrome of sudden infant death	3	0.10

Table S2. AEFI were reported in 95% (n = 2,102) of the administered vaccines.

Vaccine Type	n	Age (Mean ± SD)	Vaccines Reporting AEFI (n, %)	Number of AEFI Symptoms	Mean Number of AEFI Symptoms (Mean ± SD)
**Pfizer**	1,020	44 ± 19	965 (94.6%)	17	3.47 ± 2.54
**AstraZeneca**	833	52 ± 18	799 (95.9%)	18	3.43 ± 2.12
**Sinovac**	146	57 ± 13	135 (92.5%)	12	2.90 ± 2.15
**Moderna**	122	26 ± 8	120 (98.4%)	10	3.93 ± 1.96
**CanSino**	92	35 ± 11	83 (90.2%)	9	3.21 ± 2.13

Spearman's correlation between age and number of AEFI symptoms was statistically significant (rho= -0.163, p= 0.001), indicating that younger individuals experienced a higher number of AEFI symptoms.

Table S3. Significant associations were found between the number of AEFI reported after vaccination and age groups. The number of AEFI symptoms was higher in younger age groups.

Variable	Category	Mean (M)	SD	Test statistic (*p*)
**Sex**	Male	3.25	2.20	U = –2.002 (*p* = 0.45)
	Female	3.50	2.36	
**Age (years)**	5–9	2.44	1.94	H = 55.950 (*p* < 0.001)
	10–19	2.98	2.00	
	20–39	3.77	2.45	
	40–59	3.80	2.56	
	60–99	2.78	1.77	

Table S4. Multiple linear regression model of the variables severity of AEFI, age, sex, and number of vaccine doses

Factors	Unstandardized Coefficients β	SE	Standardized Coefficients β	t	p	95% CI for β (LL, UL)

**Model 1**						
**Constant**	1.911	0.031	—	61.850	<0.001	1.851, 1.972
**Number of doses applied**	-0.024	0.010	-0.052	-2.391	0.017	-0.044,-0.004
**Sex**	0.032	0.013	0.053	2.506	0.012	0.007, 0.056
**Age**	0.000	0.024	0.024	1.123	0.262	0.000, 0.000
**Model summary**	$R^2 = 0.085$, Std. Error = 0.260, F = 5.300, p = 0.001					
**Model 2**						
**Constant**	1.929	0.027	—	72.111	<0.001	1.876, 1.981
**Number of doses applied**	-0.036	0.010	-0.056	-2.644	0.008	-0.046, -0.007
**Sex**	0.032	0.013	0.054	2.560	0.011	0.008, 0.057
**Model summary**	$R^2 = 0.081$, Std. Error = 0.260, F = 7.319, p = 0.001					

n= 2,213, β= beta, SE= standard error, p= probability, R^2= coefficient of determination, t= Student’s t-test.
